# Mitochondria-Targeted Drug Delivery in Cardiovascular Disease: A Long Road to Nano-Cardio Medicine

**DOI:** 10.3390/pharmaceutics12111122

**Published:** 2020-11-20

**Authors:** Francesca Forini, Paola Canale, Giuseppina Nicolini, Giorgio Iervasi

**Affiliations:** 1 CNR Intitute of Clinical Physiology, Via G.Moruzzi 1, 56124 Pisa, Italy; p.canale@studenti.unipi.it (P.C.); nicolini@ifc.cnr.it (G.N.); iervasi@ifc.cnr.it (G.I.); 2 Department of Biology, University of Pisa, Via Volta 4 bis, 56126 Pisa, Italy

**Keywords:** cardiovascular disease, drug delivery, mitochondria dysfunctions, nanocarriers

## Abstract

Cardiovascular disease (CVD) represents a major threat for human health. The available preventive and treatment interventions are insufficient to revert the underlying pathological processes, which underscores the urgency of alternative approaches. Mitochondria dysfunction plays a key role in the etiopathogenesis of CVD and is regarded as an intriguing target for the development of innovative therapies. Oxidative stress, mitochondrial permeability transition pore opening, and excessive fission are major noxious pathways amenable to drug therapy. Thanks to the advancements of nanotechnology research, several mitochondria-targeted drug delivery systems (DDS) have been optimized with improved pharmacokinetic and biocompatibility, and lower toxicity and antigenicity for application in the cardiovascular field. This review summarizes the recent progress and remaining obstacles in targeting mitochondria as a novel therapeutic option for CVD. The advantages of nanoparticle delivery over un-targeted strategies are also discussed.

## 1. Introduction

Cardiovascular disease (CVD), including atherosclerosis, ischemic heart disease, hypertension, metabolic syndrome, and heart failure (HF), is a leading cause of morbidity and mortality worldwide. In most cases, the available one-target therapies relieve symptoms but do not fully address the molecular mechanisms that are often complex and interconnected.

In the last decades, mitochondria have become the fulcrum of an intense research activity aimed at developing innovative therapeutic strategies. Within the cardiovascular system (CVS), mitochondria exert key functions involved in energy production and catabolic and anabolic processes. In addition, mitochondria occupy a central position in the regulation of calcium (Ca^2+^) handling, reactive oxygen species (ROS) homeostasis, and integration of cell death or survival pathways [1,2]. Therefore, mitochondrial dysfunctions are increasingly recognized to play a major role in the pathogenesis of multiple CVDs. Alterations of mitochondrial function result in impaired electron transport chain activity with increased ROS formation, mitochondrial Ca^2+^ overload, and aberrant mitochondrial quality control, which ultimately leads to diverse forms of cell death [1,2]. In addition, damaged mitochondria trigger inflammatory responses that further contribute to the evolution of CVD.

One major determinant of the mitochondria-dependent cell loss is the opening of the mitochondrial permeability transition pore (MPTP), an unselective high conductance channel of the inner mitochondrial membrane (IMM). MTPT opening causes a collapse of the IMM potential, ATP depletion, energy crisis, and release of cytochrome c and other death factors resulting in lethal cardiomyocyte injury [2,3]. MPTP opening has been largely characterized as a key determinant of acute cardiac ischemia and reperfusion (IR) injuries but its crucial role in non-ischemic cardiomyopathy and HF has also been documented both in experimental models and in the clinical setting [4,5]. Therefore, mitochondria ROS and MPTP are primary targets for therapeutic intervention in CVD.

Attempts to limit ROS production and MPTP activation with generic antioxidants have proven ineffective in large clinical trials [6,7,8]. One of the main explanations is probably the insufficient drug delivery at the site of ROS production.

Since then, an intensive investigation effort has been dedicated to developing mitochondria-targeting agents with improved pharmacological features for preventing or treating CVD. Several drug delivery systems (DDS), based on nanocarriers, have been thus far formulated, having the ability to selectively accumulate in the mitochondria, avoiding off-target effects (Table 1).

A widely adopted strategy exploits the negative membrane potential of the IMM (Δψ_m_) to concentrate bioactive cardioprotective molecules against their concentration gradient. The most utilized mitochondria-targeting vehicle in the cardiovascular (CV) field is triphenylphosphonium (TPP), whose strong lipophilic and delocalized cationic nature has gained much traction to target various pharmacophores (Table 1). An alternative approach takes advantage of small mitochondria-targeting cationic peptides. Among them, the Szeto–Schiller-tetra-peptide 31 (SS31) and its acetate salt MTP-131 (also known as Bendavia or Elamipretide) have found widespread application in CV research (Table 1). These carriers are composed of alternating aromatic-cationic motifs (d-Arg-2′6′-dimethylTyr-Lys-Phe-NH_2_) that facilitate high solubility and uptake in a membrane potential-independent fashion [54]. Therefore, with respect to the membrane potential-dependent TPP-derivatives, SS-31 offers a greater capacity to act on both functional and diseased mitochondria.

A variety of DDSs for the treatment of CVD is based on the highly biodegradable and biocompatible poly(lactic-co-glycolic) acid (PLGA), a copolymer approved by the U.S. Food and Drug Administration (FDA) for the design of drug carriers (Table 1). Remarkably, in the setting of acute ischemic injuries, PLGA nanoparticles (NPs) ensure sustained and targeted delivery by virtue of the enhanced permeability and retention (EPR) effect that characterizes the injured tissue [43,44]. Composite polymeric PLGA delivery systems have further been developed to avoid some drawbacks associated with PLGA NPs, including poor drug encapsulation and polymer degradation. In example, coating with polyethylene glycol (PEG) copolymer is frequently adopted to form a hydrated ring that protect sensitive molecules from degradation and reduces opsonization, thus resulting in prolonged circulation time and lower degradation by the immune system [55]. Like PLGA, PEG conjugation enhances NP delivery to the injured tissue via the EPR effect. In addition to these passive targeting strategies, active drug delivery systems have also been generated by conjugating the bioactive compounds to cell-specific ligands attached to the NPs (Table 1).

Several combinations of the delivery approaches described above have been integrated in more complex formulations, leading to polymeric/lipidic NPs that are internalized via endocytosis. One of such system, called multistage continuous targeted drug delivery (MCTD), has been designed for the specific uptake of cargo by mitochondria of the ischemic myocardium [49] (Table 1). The selectivity for the ischemic left ventricle (LV) is conferred by an ischemic myocardium targeting peptide (IMTP) consisting of Ser-Thr-Ser-Met-Leu-Lys-Ala. The IMTP drives endocytosis and cellular uptake. Within the cells, the nanocarriers escape lysosome degradation and enter the mitochondria thanks to the addiction of SS31 moieties [49]. To facilitate accumulation at the ischemic tissue, the researchers also introduced PLGA and PEG modifications into the formulation (Table 1). Finally, liposome-based carriers have been developed for mitochondria delivery via membrane fusion. In one of these systems, called Mito-porter, the liposome particles carry on their surface a small octa-arginine peptide (R8) that drives cellular uptake via macropinocytosis and prevents lysosomal degradation [56] (Table 1). The lipid composition of this DDS has been designed to achieve the highest degree of fusion with the mitochondrial membrane and release of the cargo in the mitochondria [56]. A major advantage of the vesicular NPs is the ability to deliver a greater amount and wide variety of therapeutic compounds from small molecules to large macromolecules. In example, the Mito-porter system has been successfully employed to regulate mitochondrial function by driving mitochondrial uptake of several oligonucleotide species including mRNA, antisense RNA, and expression vector [50,51,52].

In light of such premises, the aim of this review is to provide an updated overview on the main mitochondria-targeted cardioprotective compounds thus far analyzed in the preclinical and clinical arena with a focus on the potentiality and pitfalls of the nanocarrier-based delivery strategies.

## 2. Mitochondria-Targeted Antioxidants

### 2.1. Coenzyme Q10 (CoQ10)

The mitochondria permeable coenzyme Q10 (CoQ10) (or ubiquinone) is the only endogenously synthesized liposoluble antioxidant. Besides its role in electron transfer chain, the fully reduced form (ubiquinol) exerts a well-characterized lipid peroxidation-inhibitory effect [57], whose therapeutic potential has largely been investigated in CVD [58].

In several animal models of acute cardiac ischemia and HF, CoQ10 treatment resulted in attenuation of mitochondria structural damage, increase of high energy substrates, improved endothelial function, and mitigation of adriamycin cardiotoxicity [58].

The clinical benefit of CoQ10 therapy is still under evaluation. If inconclusive findings have been reported in the setting of acute IR [58], a more defined cardioprotective effect has emerged with prolonged CoQ10 administration in chronic CVD. In the Q-SYMBIO randomized double blind trial, 106 weeks of CoQ10 treatment improved symptoms and reduced major adverse cardiovascular events in HF patients, while no substantial differences were observed in the short term [59]. In addition, in women with type II diabetes mellitus, long-term CoQ10 supplementation lowered major CVD risk factors including fasting blood sugar levels, insulin resistance index, and total and low density lipoprotein (LDL) cholesterol [60]. The overall data indicate a disease- and time-dependent efficacy of CoQ10 that needs to be further tested in large-scale studies. In this regard, an ongoing phase II clinical trial in a large study population is dedicated to comparison of the clinical benefits of up to 12 weeks of treatment with CoQ10 or D-ribose in patients with diastolic HF, or HF with preserved ejection fraction (HFPEF) (NCT03133793). The findings will help to better clarify the added therapeutic value of CoQ10 in HF.

### 2.2. Mitoquinone (MitoQ)

Owing to its lipophilic nature and large molecular weight, CoQ10 exhibits a rather poor bioavailability [61]. To achieve more successful mitochondria targeting and more efficient antioxidant treatment, CoQ10 has been linked to TPP vehicle to form mitoquinone (MitoQ).

Within the mitochondria, MitoQ localizes at the IMM where it acts as an effective antioxidant against lipid peroxidation and a scavenger of peroxynitrites. MitoQ treatment has been shown to confer cardioprotection in multiple in vitro and in vivo experimental models. In vitro, MitoQ prevented oxidative damage and cell death caused by hydrogen peroxide and chemically-induced IR [57,58,59,60,61,62]. MitoQ-mediated ROS scavenging was also involved in rescuing mitochondria function and cardiac performance in rodent models of acute IR [9]. In a mouse model of heterotopic heart transplantation, administration of MitoQ to the donor heart in the storage solution protected against IR injury by blocking graft oxidative damage and dampening the early pro-inflammatory response in the recipient [10]. In spontaneously hypertensive rats, 8 weeks of treatment with MitoQ reduced systolic blood pressure and attenuated cardiac hypertrophy [11]. Finally, in a mouse model of severe pressure overload, MitoQ ameliorated contractile dysfunction and improved mitochondrial network integrity and mitochondrial–sarcoplasmic reticulum (SR) alignment [12,13]. At the molecular level, MitoQ inhibited the noxious interplay between profibrotic factors and mitochondrially associated redox signaling while normalizing mitochondrial dynamics. Interestingly, MitoQ rescued the dysregulation of several redox-sensitive noncoding RNAs associated with cardiac remodeling such as the pro-hypertrophic long non-coding RNA cardiac hypertrophy-associated transcript (Chast), the anti-hypertrophic long non-coding RNA my-heart (Mhrt), and the long non-coding Plscr4/miRNA-214 axis [12,13]. Given the increasing importance of non-coding RNA species in the pathophysiology of the cardiovascular system, these findings highlight a previously unappreciated therapeutic potential of MitoQ to contrast the evolution of HF.

The promising results from preclinical studies have encouraged clinical research. In a small clinical trial in elderly subjects, 6 weeks of MitoQ treatment ameliorated age-related vascular dysfunction by improving endothelial function and reducing mitochondrial-derived oxidative stress and arterial stiffening [14]. The potential benefit of 4 weeks of MitoQ administration is also under evaluation in two ongoing clinical trials on age-dependent degenerative processes. The first pilot study will assess the effect and sex-related differences of MitoQ supplementation on mitochondrial activity, LV diastolic performance, and vascular function in elderly subjects (NCT03586414). A second phase IV study will address the role of MitoQ in ameliorating mitochondrial dysfunction, exercise intolerance, and large blood vessel hemodynamics in HFPEF patients with or without chronic kidney disease comorbidity (NCT03960073). The results of these projects may be of crucial importance to confirm the therapeutic indication of MitoQ in alleviating the age-dependent deterioration of the overall CV function.

### 2.3. 10-(6’-Plastoquinonyl)-Decyltriphenylphosphonium (SkQ1)

SkQ1 is another TPP-based lipophilic cation bearing the chloroplast-derived analogue plastoquinone instead of ubiquinone. Within the mitochondria, SkQ1 binds to cardiolipin, an IMM-specific phospholipid with a central role in the regulation of cristae architecture, respiratory chain complex integrity, and supercomplex organization [2,61]. It has been demonstrated that interaction of cardiolipin with cytochrome c converts the respiratory chain electron carrier into a peroxidase. Binding of SkQ1 to cardiolipin inhibits such a noxious interaction, thus preventing cytochrome c-mediated mitochondrial oxidative damage [15]. In several in vivo and ex vivo rat models, SkQ1 protected against hydrogen peroxide injury, cardiac arrhythmias, and myocardial infarction [16,17]. More recently, SkQ1 administration has been shown to prevent mitochondrial oxidative stress, heart hypertrophy, and diastolic dysfunction in a mouse model of high fructose-induced cardiac remodeling [18]. Moreover, SkQ1 favored antioxidant system activation in the heart and blood serum of rats with streptozotocin-induced type I diabetes mellitus [19], and alleviated heart pathology in a mouse model of premature aging [20].

SkQ1 antioxidant properties have been observed at very low concentrations, while at higher concentrations, SkQ1 becomes a prooxidant, similarly to what has been observed for MitoQ [61]. It is worth noting that the concentration range for SkQ1-induced favorable effects is greater than that of MitoQ, which may represent a potential advantage for SkQ1 implementation in the clinical arena [61].

In the clinical context, SkQ1 safety and beneficial effects have only been evaluated in patients with dry eye syndrome [63]. Given the high antioxidant property at a low dose observed in preclinical studies, testing the therapeutic potential of SkQ1 in patients with CVD may open new therapeutic perspectives.

### 2.4. Mito-Tempo (MT)

Mito-Tempo (MT) is a mimetic of the mitochondrial superoxide dismutase consisting of a TPP-conjugated piperidine nitroxide. Besides detoxifying superoxide radicals, it also limits hydroxyl radical production by oxidizing ferrous iron [64]. The protective effects of MT have been documented in a wide range of experimental animal models. In a rat setting of metabolic syndromes, elimination of the mitochondrial oxidative stress with MT rescued cardiac collateral growth after repetitive ischemia insults [21]. MT was also shown to attenuate cardiomyopathy and preserve cardiac function in streptozotocin and db/db mouse models of type I and type II diabetes as well as in high-fat diet-fed mice [22,23,24]. In mouse models of renin–angiotensin system activation, MT reduced cellular superoxide and improved endothelial function and NO bioavailability by decreasing nicotinamide adenine dinucleotide phosphate (NADPH) oxidase [25,26,27]. In these contexts, MT prevented ventricular tachycardia and sudden cardiac death by increasing the connexin-43 levels and gap junction organization [25,26,27]. Interestingly, MT was able to reduce blood pressure even after the onset of hypertension while non-mitochondrial targeted antioxidants were ineffective [25,26,27]. Similarly, in a HF mouse model with cardiac-restricted mitochondrial calpain overexpression, MT but not general antioxidants attenuated cardiac cell death, HF evolution, and mortality [28]. In the murine model of chronic pressure overload MT administration markedly improved both cardiac contractile performance and mitochondrial respiratory function by decreasing the oxidative post-translational modification to Lon protease homolog, a mitochondrial protease critically involved in the process of mitochondria quality control [29]. Finally, the cardioprotective action of MT was recently extended to a more clinically relevant pig model of severe pressure overload and β-adrenergic stimulation that better recapitulated important features of non-ischemic arrhythmogenic HF [30]. In such a large animal model, chronic administration of MT prevented HF and effectively reduced ROS production in both mitochondrial and cytosolic compartments of the failing myocytes, which was associated with the normalization of the perturbed mitochondrial proteome and phospho-proteome [30]. Analogous to the above-mentioned hypertension mice models, MT was also effective after the onset of cardiac hypertrophy, suggesting that MT administration can be exploited even to revert overt cardiac disease [30].

Accordingly, in an ex vivo study on arterioles and mononuclear cells from patients with type II diabetes, MT exposure significantly reduced mitochondrial ROS and improved endothelial and vascular function [31].

**Figure 1:**
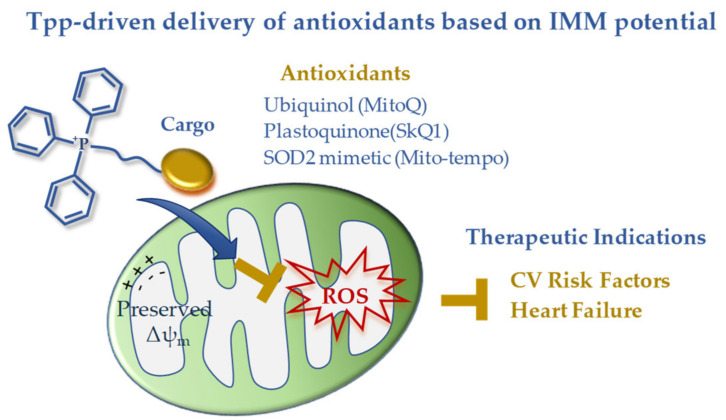
Mitochondria-targeted delivery of antioxidants based on triphenilphosphonium lipophilic cation.

In summary, the available clinical data on TPP-derived antioxidants indicate a disease-dependent efficacy that is expected to guide future development (Figure 1). The disappointing results in acute ischemic insults are counterbalanced by the effectiveness of long-term treatments in reverting risk factors and chronic/degenerative CV alterations leading to HF progression. The disease-dependent behavior of these drug carriers is consistent with a different maintenance of the IMM potential that abruptly falls in the ischemia-reperfused cell, thus exhausting the driving force for lipophilic cation uptake. In the presence of better preserved IMM potential, such as in non-ischemic CVD, TPP-linked scavenging compounds could represent good candidates to overcome the permeability and targeting problems observed with general antioxidants (Figure 1). However, the clinical data are still very limited, and more work on large patient cohorts is needed in order to better understand the therapeutic efficacy of this mitochondria-targeting strategy.

### 2.5. Szeto–Schiller 31 (SS31)

Szeto–Schiller 31 (SS-31) and its acetate salt MTP-131 (also known as Bendavia or Elamipretide) are highly permeable mitochondria-targeted antioxidant peptides. SS31 was initially thought to exert its protective effect mainly through the ROS-scavenging activity of the dimethyltyrosine residue [65,66]. However, subsequent studies revealed a further mechanism of SS31 action. Similarly to SkQ1, SS31 binds to cardiolipin and modulates its hydrophobic interaction with cytochrome c to block the peroxidase activity of the carrier in favor of the electron transfer function [54,67]. More in depth insights on the SS31 activity within the cardiac mitochondria have been provided in a recent mass spectrometry analysis of the SS31-protein interaction landscape [68]. The identified SS31 interactors, all cardiolipin-binding proteins, play a key functional role in ATP production and transport, as well as in 2-oxoglutarate metabolism and signaling, which is consistent with improved mitochondrial function resulting from SS31 treatment [68].

The cardioprotective effect of SS31 has been documented in several experimental settings of cardiac disease. In small and large animal models of cardiac IR, the administration of SS31, either at the onset of ischemia or even just prior to reperfusion, limited myocardial damage and adverse remodeling and improved the recovery of cardiac function [69,70,71]. SS31 has also been shown to protect against pathological hypertrophy and fibrosis in mice models of hypertensive cardiomyopathy or transverse aortic constriction (TAC)-induced pressure overload [68,69]. Such beneficial effects were paralleled by reduced degree of mitochondria functional and structural alterations and by amelioration of proteomic profiling of either mitochondrial or non-mitochondrial proteins [72,73]. In a dog model of microembolism-induced HF, SS31 chronic treatment following the onset of HF preserved mitochondria function and bioenergetic, decreased ROS burden, improved systolic function, and reduced the plasmatic biomarkers of HF [74]. Finally, in a pig model of metabolic syndrome, SS31 administration attenuated the early alterations to cardiac function to cardiac mitochondria organization and to mitochondria–SR interaction [75].

Overall, these preclinical studies confirm that mitochondrial dysfunction and oxidative damage are crucial pathogenic mechanisms in CVD and that pharmacological interventions to preserve cardiolipin and cardiolipin–protein interactions would be of great therapeutic relevance even to revert overt pathologies.

However, translating the benefits of SS31 to the bedside has proven challenging either in acute ischemia and HF. The EMBRACE STEMI multicenter phase IIa trial evaluated the efficacy of SS31 in 297 patients with ST-segment elevation myocardial infarction (STEMI) [76]. While safe and well tolerated, SS31 infusion started before the onset of percutaneous coronary intervention and lasting for 1h after reperfusion, showed no significant impact on infarct size or LV function [76].

In the setting of HF with reduced ejection fraction (HFREF), a preliminary success was obtained in a proof-of-concept phase I randomized, placebo-controlled trial [77]. In this study, patients were treated with a single 4 h intravenous infusion of SS31 at different dosages with a 24h follow-up. The high-dose SS31 induced a significant decrease in LV end diastolic (LVEDV) and end systolic volumes (LVESV), suggesting that SS31 could improve cardiac function in HF. Of note, the maximal cardioprotective effect was observed at the peak of SS31 plasma concentration and decreased as the plasma concentration of SS31 disappeared (24h post-infusion). These findings highlight an important dose–effect relationship that is critically influenced by SS31 plasma half-life [77]. The positive results were not confirmed in a successive phase II clinical trial on HFREF patients. Although well tolerated, 4 weeks of daily subcutaneous infusion of SS31 did not produce the expected effects on primary and secondary endpoints including LVESV, Nt-proBNP plasma levels, and LV ejection fraction [78]. As a partial explanation, it has been postulated that 4 weeks treatment may be insufficient to correct cardiac remodeling and mitochondrial dynamics in HF [78]. These disappointing findings highlight the need to elucidate the optimal dosing, timing, and duration of drug administration to successfully move from the bench to the bedside. In addition, with SS31 being a cationic peptide, there may be some factors limiting its bioavailability in human myocardial mitochondria such as systemic proteases, rapid metabolism, and sequestration by blood products, which ultimately may result in a short half-life. The rapid clearance of uncojugated SS31 from plasma observed in patients supports this hypothesis [77]. In addition, direct incubation of freshly isolated myocardial mitochondria with SS31 effectively improved the organelle function and rescued the altered activity of supercomplexes CI, CII, and CIII [79], thus confirming that the negative results of clinical trials may have arisen, at least in part, from insufficient mitochondrial targeting within the cardiac tissue.

**Figure 2:**
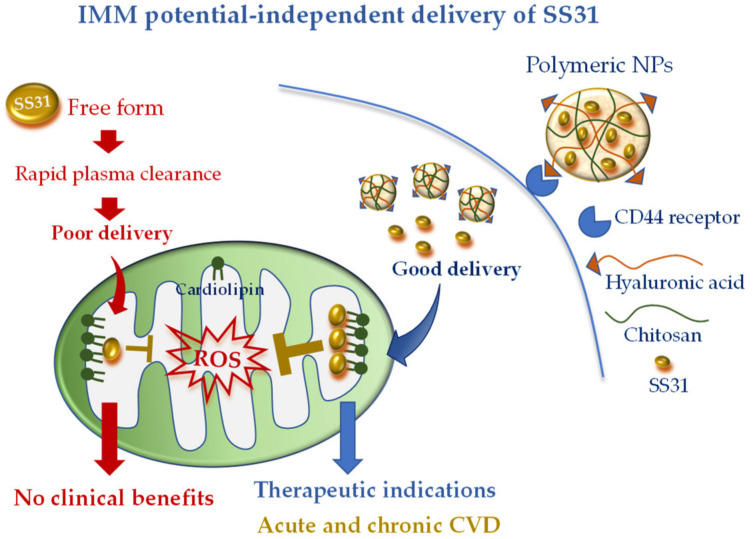
Mitochondria-targeted delivery of SS31 in free form versus nanoparticle (NP) formulation. Free SS31 is more prone to plasmatic degradation and rapid clearance, which might have undermined clinical benefit. NP formulation, with cell targeting moiety (hyaluronic acid) and higher mitochondrial uptake, is expected to confer promising therapeutic efficacy either in acute or chronic cardiovascular disease (CVD).

To overcome this limitation, Liu et al. [42] encapsulated SS31 in pH-sensitive NPs composed of cationic chitosan, a linear polysaccharide used for drug delivery [80], and anionic hyaluronic acid (HA), a compound with known selectivity for the receptor CD44. It is worth noting that CD44 is upregulated at the site of inflammation and cell injury [77,81]; therefore, this active DDS possesses diverse advantages: (i) the ability to protect SS31 from blood proteases, (ii) the ability to rapidly release SS31 in acidic pH conditions, and (iii) more specific cell targeting (Figure 2). The combination of such cell-targeted and organelle-targeted NP formulation has been successfully validated in human endothelial cells subjected to oxidative stress and in a rodent model of acute renal injury induced by ROS [64] (Figure 2).

Since CD44 is also overexpressed in acute and chronic CVD [81,82,83], the new formulation appears as a promising approach to concentrate the antioxidant and anti-inflammatory properties of SS31 at the site of cardiac injury. Of note, owing to the IMM potential-independent uptake of SS31, this DDS may also be ideal in particularly injured mitochondria such as in myocardial IR. Further studies are recommended to validate such an interesting hypothesis.

## 3. Inhibition of MPTP Opening

Another major target of cardioprotection is the mitochondrial permeability transition pore (MPTP) [84]. Although the molecular nature of the MPTP is still controversial, the matrix protein cyclophilin D (Cyp D) plays a crucial role in MPTP opening upon heart injury and has long been regarded as a target for limiting cell loss and improving heart performance in the post-ischemic setting [85,86]. Cyclosporin A (CsA), a lipophilic cyclic peptide initially developed as an immunosuppressant, has also been demonstrated to inhibit MPTP opening by binding to CypD [87]. Several experimental studies in small and large animal models have documented the myocardial infarct size-limiting properties of CsA administered at the time of reperfusion, when the MPTP opening is expected to occur [85]. CsA has also been reported to protect the heart in other experimental settings of acute IR injuries such as neonatal cardioplegic arrest and reperfusion and resuscitated cardiac arrest [85].

In accordance with the animal studies, CsA administration at the time of reperfusion has been shown to protect the heart against IR injury in patients undergoing aortic valve surgery [88] and to reduce infarct size and circulating injury biomarkers in a small proof-of-concept clinical trial on STEMI patients [89]. In contrast to the encouraging preliminary results, in the successive CIRCUS and CYCLE large multicenter clinical trials on STEMI patients, a single bolus CsA administration was unable to show any benefit on a variety of endpoints such as ST segment resolution, LV function, adverse remodeling, release of cardiac damage biomarkers, and clinical outcomes at 6 or 12 months [90,91]. Matching the results of the CIRCUS and CYCLE studies, in a more recent and smaller trial (CAPRI), a single CsA bolus did not affect infarct size or LV remodeling in STEMI patients [92]. Moreover, the characterization of the lymphocyte kinetics and plasmatic concentration of CsA during the 24 h period post-injection offered some putative explanations of the negative results: (i) CsA did not reach ischemic cardiomyocytes, (ii) CsA needed to be given at an earlier time point during myocardial ischemia, and (iii) 1 bolus of CsA is not sufficient to inhibit the proliferation of the pro-inflammatory CD4 T-lymphocyte during remodeling. Such indications may help to draw the direction of future studies.

**Figure 3:**
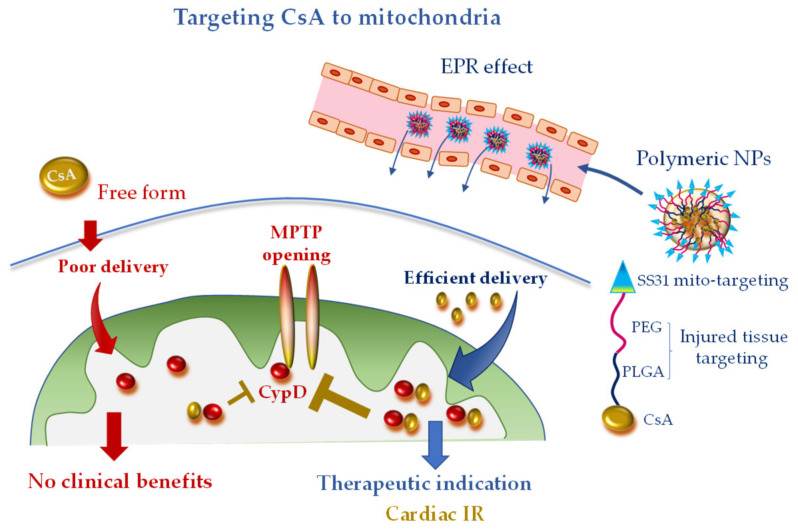
Mitochondria-targeted delivery of cyclosporine A (CsA) in free form versus nanoparticle (NP) formulation. Free CsA suffers of poor delivery at the injured tissue, which might have undermined clinical benefit. NP formulation, with tissue injury targeting moieties (polyethylene glycol (PEG) and poly(lactic-co-glycolic) acid (PLGA)) and mitochondriotropic SS31 moiety, is anticipated to confer promising therapeutic efficacy, especially in acute ischemic CVD. CydD, cyclophillin D; EPR, enhanced permeability and retention; IR, ischemia and reperfusion; MTPT, mitochondrial permeability transition pore; PEG, polyethylene glycol; PLGA, poly(lactic-co-glycolic).

Recently bioabsorbable PLGA-CsA NPs have been developed to improve the bioavailability and facilitate delivery of CsA to the injured cardiomyocyte, thus obtaining a more efficient inhibition of MPTP opening. The accumulation of PLGA-CsA NPs at the IR myocardium is favored by the EPR effect at injured tissue [43,44]. In line with the expectancy, in cardiomyocyte cultures, PLGA-CsA NPs demonstrated a greater propensity to accumulate in ROS-injured mitochondria and an improved cytoprotection at a dose lower than CsA alone [43]. In a murine model of IR, CsA loaded in PLGA NPs showed a more enhanced cardioprotective effect than CsA alone through the inhibition of MTPT opening [43]. However, PLGA-CsA NPs may also present unfavorable features such as the unspecific uptake by the mononuclear phagocyte system in the blood circulation, which is expected to reduce the accumulation of PLGA-CsA NPs in mitochondria of the ischemic cardiomyocytes [44,93]. To address this constraint and avoid extra-mitochondrial accumulation of CsA, researchers have formulated hybrid PLGA-CsA NPs by coating PEG on PLGA NP surface. Moreover, the mitochondriotropic SS31 peptide has been added to the formulation to increase mitochondrial delivery [43] (Figure 3). The resulting CsA-PLGA-PEG-SS31 DDS exhibited significant cardioprotective effects against IR in rats through accumulating in the injured mitochondria, protecting mitochondrial integrity and decreasing cardiomyocyte cell death and myocardial infarct area [43]. Alternatively, Mito-porter could potentially be used to deliver mitoprotective agents such as CsA to cardiac mitochondria in ischaemic cardiomyocytes following acute myocardial infarction [53].

These promising nanocarrier approaches may have great therapeutic potential to be tested in future large animal models of CVD and pilot clinical studies.

## 4. Mitochondria-Targeted Donors of Nitric Oxide (NO) and Hydrogen Sulfide (H_2_S)

Nitric oxide (NO) and hydrogen sulfide (H_2_S) at low concentrations exert well-documented cardioprotective actions against IR injuries by acting as signaling molecules and by inducing redox-based post-translational modifications on thiol groups of key mitochondrial proteins [94,95,96,97,98]. As a consequence, mitochondrial-targeted exogenous donors of NO or H_2_S have been formulated as powerful tools for basic studies and innovative pharmacotherapeutic agents in ischemic cardiac disease (Table 2).

### 4.1. MitoSNO

The mitochondria-selective S-nitrosating agent (MitoSNO) is a TTP-linked S-nitrosothiol that selectively modulates and protects mitochondrial function from excessive ROS formation at the onset of myocardial reperfusion (Table 2). In several rodent models of IR, MitoSNO has been shown to limit infarct size, post-ischemic adverse cardiac remodeling, and HF evolution [32,33,34,35]. Mechanistically, MitoSNO transfers a nitric oxide moiety onto particular thiol proteins on respiratory complexes I and IV. In particular, selective S-nitrosation of complex I slows the reactivation of mitochondria during the crucial first minutes of the post-ischemic tissue reperfusion, thereby decreasing ROS production, oxidative damage, and tissue necrosis [32,34]. The study on MitoSNO-derived S-nitrosation in IR identified a number of other enzymes of central importance for mitochondrial metabolism, specifically those supplying electrons to the respiratory chain from the breakdown of carbohydrates and fatty acids [33]. The reversal of such post-translation modifications 5–10 mins after the onset of reperfusion is supposed to allow the mitochondria to return to full activity in a more physiological context and confer long-lasting protection against post-ischemic HF development [32,33,34,35].

### 4.2. AP39

AP39 is a mitochondrial-targeted, TPP-bound donor of H_2_S, with cardioprotective effect documented either in vitro and in vivo (Table 2). AP39 has been shown to protect endothelial cells from hyperglycemia-induced mitochondrial-derived oxidative damage and has been proposed as a helpful agent against diabetic vascular complications [36]. In cardiomyocyte cultures, AP39 inhibited ROS-dependent mitochondria injuries and cell death by increasing the mitochondrial Ca^2+^ retention capacity and directly inhibiting MPTP opening [37]. In a mouse model of cardiac arrest and cardiopulmonary resuscitation, AP39 administration at the onset of resuscitation improved neurological outcomes by maintaining mitochondrial integrity and reducing oxidative stress [38]. In mice models of IR, AP39 injected at reperfusion provided direct mitochondria and cardiac protection through inhibition of MPTP opening at a site different than CypD and independently from the activation of cytosolic pro-death pathways [37,39]. In a recent work on mice, supplementing AP39 in the preservation solution protected cardiac grafts from prolonged ischemia, highlighting the therapeutic potential of this approach in preventing IR injury in heart transplant [40].

### 4.3. Isothiocyanate Derivatives

Isothiocyanate derivatives have also been synthetized as mito-protective H_2_S-donor compounds (Table 2).

The 4-carboxy-phenyl-isothiocyanate (4CPI) is a slow H₂S-releasing molecule, endowed with vasorelaxant and hypotensive effects [99]. In isolated rat hearts subjected to IR, preconditioning with 4CPI inhibited ROS formation, improved the post-ischemic recovery of myocardial functional parameters, and limited tissue injury [100]. These effects were antagonized by a specific blocker of the mitochondria ATP-sensitive potassium channel (mitoKATP), a recently identified protein of the IMM, whose activation under anoxic condition exerts a series of well-documented cardioprotective actions [101,102].

In a similar work, 3-pyridyl-isothiocyanate (3PI) has been identified as a new promising cardioprotective agent by means of an isothyocianate library screen [103]. The beneficial pharmacological properties were successfully characterized in ex vivo and in vivo rat IR models in which 3PI administration before the IR procedure resulted in significant reduction of infarct size and attenuated the release of injury biomarkers [103]. The antioxidant and antiapoptotic responses to 3PI were largely dependent by the activation of mitoK-ATP channel, thus confirming the crucial involvement of this channel in the cardioprotective effects of isothiocyanate derivatives.

The clinical benefits of H_2_S donors in IR patients has never been investigated. With the exception of MitoSNO, a major limitation of these compounds is the characteristic to confer the best protection when administered as a preconditioning strategy before the ischemic insult. A more feasible application of H_2_S donors against IR injuries in patients could be supplementation in the cardioplegic solution during heart surgery or in the preservation medium before heart transplant (Table 2). As a final point, H_2_S donors have been investigated only in the setting of IR; however, a recent finding opens novel possibilities extending the potential therapeutic effect of this strategy also to overt HF [104]. Further preclinical and clinical studies are indispensable to fix these critical aspects.

## 5. Inhibitors of Mitochondrial Fission

Altered mitochondrial morphology, with increased fission and fragmented mitochondria, is a hallmark of mitochondrial dysfunctions in a variety of human CVD, including acute ischemia and HF [105,106]. Since the GTPase dynamin-1-like protein (Drp1) is one main executor of mitochondria fission, specific inhibitors of Drp1 have been developed and tested in preclinical animal models of cardiac disease.

### 5.1. Mitochondrial Division Inhibitor 1 (Mdivi-1)

Mitochondrial division inhibitor 1 (Mdivi-1) is a quinazolinone derivative identified as a Drp1-selective inhibitor through a chemical library screen. Mdivi-1 has been firstly reported to inhibit Drp1-dependent mitochondrial fission, cytochrome c release, and apoptosis in yeast and non-cardiomyocyte mammalian stressed cells [107]. In in vitro and in vivo murine models of cardiac IR, Mdiv-1 prevented mitochondria fragmentation and dysfunction, limited cell death, attenuated the incidence of arrhythmia, and reduced infarct size [106,108,109]. In these studies, Mdivi-1 administration was protective either before or during the ischemic insult, even though the best results were obtained with the pre-ischemia treatment [106]. On the contrary, in a small pilot study on a more clinically relevant pig model of IR, administration of Mdivi-1 immediately prior to the onset of reperfusion did not reduce infarct size or preserve LV function [108]. The authors concluded that larger studies with different Mdivi1 dosages and more specific Drp1 inhibitors are required before translating the benefit of Drp1 targeting to patients.

The cardioprotective effects of Mdivi-1 have also been explored in non-ischemic settings of adverse cardiac remodeling [110,111]. In spontaneously hypertensive rats and mice with pressure overload-induced HF, daily injection of Mdivi for 7/8 days improved LV function and reduced the extent of myocardial fibrosis and cell death by mitigating mitophagy due to excessive mitochondrial fission [110,111].

To the best of our knowledge, thus far, human studies are limited to Mdivi-1 application in in vitro or ex vivo settings. In human endothelial cells in culture and in human arterioles, Mdivi-1 prevented mitochondria fragmentation and improved vascular function after a clinically relevant low-glucose exposure [112]. On the basis of these data, it has been suggested that Drp1 may represent a therapeutic target for improving cardiovascular complications among diabetic patients receiving intensive glucose control therapy [112]. Worthy of mention, Midivi1 also promoted cardiac differentiation of human induced pluripotent stem cells (iPSCs) in culture by shifting the balance of mitochondrial morphology toward fusion. According to these findings, Drp1 may represent a new molecular target to promote the differentiation of human iPSCs into cardiac lineages for future personalized cardiac-regenerative medicine [45].

**Figure 4:**
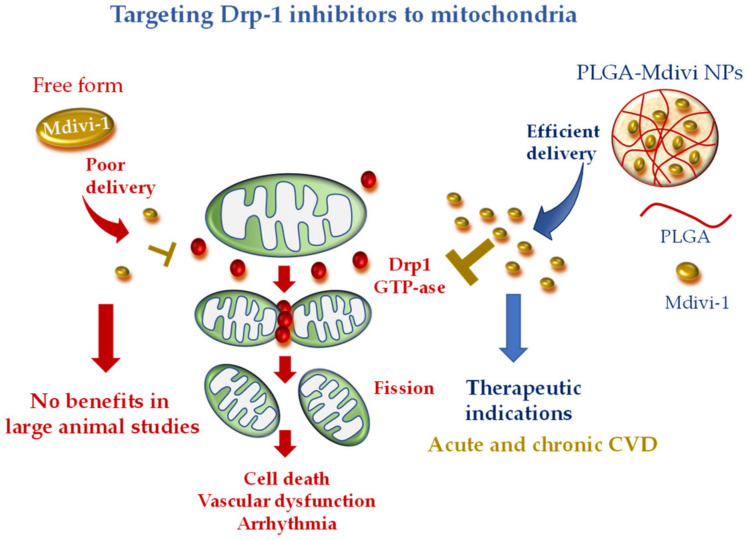
Mitochondria-targeted delivery of dynamin-1-like protein (Drp1) inhibitor (mitochondrial division inhibitor 1 (Mdivi-1)) in free form versus nanoparticle (NP) formulation. Free Mdivi-1 may suffer from poor delivery at the injured tissue in clinically relevant large animal models, which might abrogate the benefits observed in small animals. NP formulation, with tissue injury targeting moieties, is anticipated to confer promising therapeutic efficacy either in acute or chronic CVD.

Recently, a more precise mitochondrial-targeted delivery of Mdivi1 was developed by loading the drug in PLGA NPs [113]. Midivi1-PLGA NPs better protected rat neonatal cardiomyocytes against H_2_O_2_-induced oxidative stress in comparison with Mdivi-1 alone. The improved mitochondrial localization and greater beneficial effects of Midiv1-NP were also confirmed in Langhendorff and in vivo mouse models of cardiac IR treated with the DDS at the time of reperfusion [113]. These results raise the interesting working hypothesis that Midivi1 NPs may be used to overcome the limitation of un-targeted Midiv1 observed in large animal studies, shortening the road to clinical translation (Figure 4).

### 5.2. Drp1 inhibitor 1 (Driptor1) and Drp1 inhibitor 1a (Driptor1a)

Novel Drp1 inhibitors have been recently identified by an in silico chemical screen. Among them, the ellipticine compound, termed Drp1 inhibitor 1 (Driptor1), and a congener of Driptor1, termed Drp1 inhibitor 1a (Driptor1a), were demonstrated to exert a more potent and specific Drp1 inhibitory effect than MDVI-1 [114]. In particular, Driptor1a offered cardioprotection in a rat Langendorff right ventricle IR model [114]. The full potential of these very preliminary findings needs to be further explored in small and large animal models of CVD.

## 6. Mitochondria-Targeting of Natural Compounds with Pleiotropic Effects

**Figure 5:**
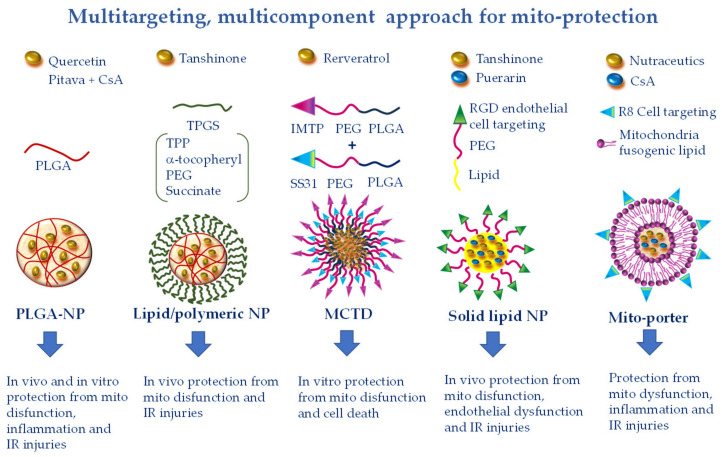
Multitargeting, multicomponent approaches for mito-protection exploiting the pleiotropic effects of several nutraceutics or the combination of more protective compounds with different mechanisms of action. NPs can be functionalized to achieve optimal targeting of specific tissue districts and cell types. Polyethylene glycol (PEG) and poly(lactic-co-glycolic) acid (PLGA) accumulate NPs at the injury site because of the enhanced permeability and retention effect. The ischemic myocardium-targeting peptide (IMPT) ensures vehiculation at the IR zone. The cell-targeting peptides RGD and R8 drive cell uptake. Finally, triphenilphosphonium (TPP), Szeto–Schiller 31 (SS31), and mitochondria fusogenic lipid moieties elicit greater mitochondrial uptake.

Cardiovascular diseases are complex multifactorial pathologies in which multiple components of mitochondrial physiology in different cell types concur to speed up the disease evolution. Therefore, strategies directed at a single target may be insufficient to ensure adequate protection [115]. Different natural bioactive compounds have thus far been described with pleiotropic beneficial effects on a plethora of CVD [116]. Among them, phenolic and terpenoid phytochemicals have been shown to protect the vascular and cardiac function against mitochondria-mediated pro-oxidant, pro-apoptotic, and pro-inflammatory injuries through the modulation of multiple cell signaling transduction pathways in different cell types [116]. Thanks to their multitargeted effects and good tolerability, natural molecules are promising candidates to counteract the development of multifaceted disorders such as CVD (Figure 5).

### 6.1. Resveratrol

Resveratrol (RES), a polyphenol contained in abundance in grape skins, exhibits attractive antithrombotic, anti-inflammatory, and antioxidant properties to be exploited in the context of acute and chronic CVD [117]. Increasing experimental evidence have demonstrated the cardioprotective effects of RES against LV dysfunction and adverse remodeling following IR injury, pharmacological agent-induced cardiotoxicity, obesity, and diabetes [118]. Mainly, RES improves vascular and cardiac performance via protection of endothelial and cardiac mitochondrial function [119,120]. Its beneficial effects largely derive from the intrinsic ROS scavenging activity, the ability to increase antioxidant defense, and the ability to modulate cytokine production and mitochondrial biogenesis [117,121].

More contrasting results have been reported in clinical studies [121]. Despite good tolerability and protective effects, especially at higher doses, available human studies indicate a rapid metabolism of RES, which may have limited its cardiac bioavailability in some cardiac patient cohorts [122,123].

This constraint has been addressed by a nano-formulation recently developed to specifically target resveratrol to cardiac mitochondria. In this study, resveratrol was delivered in the vesicular multistage continuous targeted drug delivery NPs (MCTD-NPs) either in rat cardiomyoblasts in culture or in a rat model of cardiac IR [49]. Thanks to the IMTP moiety, the intracellular uptake of MCTD-NPs was specifically enhanced in IR injured cells with a concomitant reduction of mitochondrial ROS, MPTP opening, and mitochondria-dependent apoptotic pathways (Figure 5). In vivo, MCTD-NP administration at the onset of reperfusion increased the distribution of RVS in the ischemic myocardium and reduced infarct size with an increased efficiency with respect to RVS alone or RVS delivered in PLGA NPs [49]. These results demonstrated the reliability of a novel platform for specific delivery of protective cargo to cardiac mitochondria in the setting of ischemic cardiac disease.

### 6.2. Quercetin

Quercetin (QUE) is a polyphenol extracted from various plants. Experimental in vivo and in vitro studies have shown that quercetin has a wide range of biological actions including anti-inflammatory activities as well as the ability to attenuate oxidative stress, lipid peroxidation, platelet aggregation, and capillary permeability [124,125]. Furthermore, QUE has a prominent protective action against mitochondrial dysfunction and mitochondria-dependent cell death [126,127]. In accordance, a meta-analysis of clinical trials evidenced a blood pressure-lowering activity of high dosage QUE intake [128]. 

Similarly to RES, QUE presents pharmacokinetic hurdles, with only 20% of the administered dose reaching the blood. To improve QUE bioavailability, NP-mediated delivery has been implemented. In a recent work, QUE was encapsulated in PLGA-NPs and tested in vitro in a surrogate model of cardiac cells [46]. The higher delivery degree of encapsulated QUE with respect to free QUE resulted in a superior protection capacity, as evidenced by improved antioxidant properties, decreasing cell death after IR injury and preserving mitochondrial membrane potential and ATP synthesis (Figure 5). The results point to the potential of this strategy for the treatment of oxidative stress in cardiac diseases [46]. Further works are necessary to confirm these findings in an in vivo model of CVD.

### 6.3. Isosteviol

Isosteviol (IST) is a bioactive diterpenoid extracted by *Stevia rebaudiana* that has a variety of biological activities targeted at the CVS, including anti-hypertensive, anti-hyperglycemic, antioxidant, and anti-inflammatory effects [129]. In cardiomyoblasts subjected to simulated IR or pro-hypertrophic injuries, IST restored mitochondrial membrane potential, morphological integrity, and biogenesis; decreased ROS levels; and upregulated the expression of antioxidant enzymes [130,131]. In addition, IST relieved IR injury in rodent hearts and isolated pig hearts [132,133]. At least some of the observed beneficial effects of IST can be attributed to stimulation of the mitoKATP channel, since a selective mitoKATP inhibitor abolished its protective action [133].

The key role of mitoKATP channels in the IST cardioprotective profile suggested a strategy for effectively driving diterpene compounds into the mitochondria to improve their pharmacokinetic profile and, consequently, their pharmacological effects. The mitochondriotropic properties of a TPP conjugate formulation of IST have been investigated in vitro and in vivo [41]. In a heart cell line, the mitochondrial uptake of TPP-IST was associated to mild IMM depolarization and inhibition of Ca^2+^ overload, which is compatible with activation of mitoKATP channel [41]. Administration of TPP-IST to a rat model of IR exerted significant cardioprotective effects at a 100-fold lower concentration with respect to the effective dose of free IST, suggesting that the mitochondrial delivery afforded by the TPP strategy led to a significant improvement of the cardioprotective effects [41].

### 6.4. Tanshinone

Tanshinone (TN) diterpene compound is a major active ingredient derived from the Chinese medical herb *Salvia miltiorrhiza* and is a widely investigated therapeutic agent for the treatment of CVD [134]. Thanks to its pleiotropic antioxidant, antihypertensive, anti-inflammatory, and lipid lowering activities, TN inhibits cardiac IR injury and adverse remodeling, blunts endothelial and vascular dysfunctions, and prevents platelet aggregation [134]. Its main mechanisms of action are inhibition of mitochondrial ROS production, MPTP opening, and mitochondria-mediated cell death. However, its poor water solubility and low oral bioavailability have hindered its clinical application.

To overcome this limitation, a lipid-polymeric nanocarrier (LPN) for mitochondrial-targeted delivery of TN has been recently developed. The formulation consists in a PLGA-TN mixture enclosed in a lipophilic shell formed by TPP linked to a D-α-tocopheryl-PEG-succinate (TPGS) moiety, an FDA-approved biocompatible excipient widely used for drug delivery [135] (Figure 5). The TN-LPN exhibited a better efficiency in terms of compatibility, biodistribution, and pharmacokinetic profile with respect to free TN and PLGA-TN NP formulations. It is worth noting that evident cardioprotective effects were observed in a rat model of IR, in which TN-LPN was added at the onset of reperfusion [48].

These results indicated that the TPP-TPGS/TN/LPNs represent promising nanocarriers for efficient delivery of cardiovascular drugs and other therapeutic agents for the treatment of CVD. However, future studies are needed to better evaluate the safety and efficacy of such an approach in different CVD settings and in large animal models.

## 7. Simultaneous Drug Delivery for a More Efficient Combination Therapy

Another promising multi-component and multi-targeted approach consists in the combined delivery of more than one cardioprotective agent. In a recent study by Gao et al., solid lipid nanocarriers made of DSPE (1,2-distearoyl-sn-glycero-3-phosphoethanolamine) were co-loaded with TN and puerarin (PUE)-prodrug [136] (Figure 5). PUE is a major active ingredient derived from the Chinese medical herb *Radix puerariae*, with significant mito-protective effects directed at the endothelial cells [137]. To favor a more precise targeting of PUE to endothelial cells of the ischemic myocardium, vesicular NPs have been developed with PEG-modified cyclic arginyl-glycyl-aspartic (RGD) acid peptide. The PEG particle drives the accumulation at the infarct site due to the EPR effect, while the RGD moiety is a specific ligand for the endothelial avb3 integrin receptor. This DDS has proven effective in reducing infarct size in a rat model of acute myocardial infarction [138]. The same approach used for the simultaneous administration of TAN and PUE resulted in greater cellular uptake and smaller infarct size with respect to the single phytochemicals delivered either in free or NP formulations [136]. The findings indicate the synergistic effect of the double drugs loaded in one system, suggesting a promising strategy for the treatment of myocardial infarction.

Along the same line, in another work, PLGA-based polymeric NPs containing CsA (CsA-NPs) and pitavastatin (Pitava-NPs) were simultaneously administered to target mitochondrial dysfunction and monocyte-mediated inflammation in a mouse model of acute cerebral IR [47]. Through blocking MPTP opening and chemokine receptor-2-dependent inflammation, concomitant administration of CsA-NPs and Pitava-NPs at the time of reperfusion decreased infarct size and attenuated neurological deficits as compared to single administration of CsA-NPs or Pitava-NPs (Figure 5). Given the crucial involvement of MPTP opening and inflammation in promoting cardiac injuries, it is conceivable that a similar NP-based combination therapy could also provide benefits in CVD [47]. Finally, the highly versatile Mito-porter DDS can potentially be employed to achieve mitochondria-targeted multiple delivery of protective agents, including nutraceutics and CsA, for a more efficient combination therapy in CVD (Figure 5).

## 8. Conclusions and Future Perspectives

As the central role of mitochondrial signaling in CVD has become clearer, research efforts have been oriented toward direct modulation of mitochondrial functions. CoQ10, SS31, and CsA are the better characterized unconjugated mito-drugs thus far tested. However, despite encouraging results emerging from animal models, no mitoprotective drugs have passed clinical trials in large patient cohorts [6,7,8,76,90,91,92]. One possible explanation may have been poor delivery to the diseased cells and tissue districts. In this regard, nanotechnology has made huge progress in recent years to address the current limitations and to offer sustained delivery to mitochondria. Nanopreparations can be optimized to achieve improved biodegradability, pharmacokinetic properties, and bio-distribution profiles.

A variety of promising new cardiovascular nanoformulations have been tested in in vitro and in vivo experimental models. Although exciting, most of these studies are still at an embryonic preclinical stage. On the road to nano-cardio medicine, several critical issues need to be addressed to accomplish a more realistic translatability to human health. The vast majority of experimental studies have been performed in small-sized young animals without comorbidities, which hardly recapitulates the conditions of elderly cardiovascular patients presenting with more than one pathology. Therefore, a more rigorous design of pre-clinical models, with accurate selection of dosage and mode of administration and taking into account aging, sex differences, comorbidities, and co-medications, is of paramount importance for the development of more successful clinical trials. As a second key point, efficacious DDS must face the multifactorial nature of CVD. Innovative therapeutic strategies should comprise a multi-faceted approach targeted at different mitochondrial noxious pathways, without disregarding non-cardiomyocyte cells including fibroblasts, endothelial cells, and inflammatory cells that critically contribute to CVD evolution. The emerging multifunctional, mitoprotective NPs might represent good candidates to enable such a paradigm shift in the future (Figure 5).

## Figures and Tables

**Table 1:** Main delivery strategies and protective cargos explored in the cardiovascular field.

Carrier	Delivery Strategy	Cargo	Cardioprotective Effect
TTP small molecules (lipophilic cation)	Negative IMM potential	Antioxidants, H_2_S and NO donors, isosteviol	ROS scavenging [9,10,11,12,13,14,15,16,17,18,19,20,21,22,23,24,25,26,27,28,29,30,31] PTM [32,33,34,35,36,37,38,39,40] mitoKATP activation [41]
PH-sensitive polymeric NPs (chitosan, HA)	CD44-dependent cell targeting, SS31(Mito-targeting)	SS31	Cardiolipin binding [42]
Polymeric NPs (PLGA; PEG; PEG-PLGA)	EPR effect (injured tissue-targeting), RGD (endothelial cell targeting), SS31 (mito-targeting)	CsA, fission inhibitors, nutraceutics	Cyclophylin D binding [43,44], inhibition of MPTPO [45], pleiotropic [46,47]
Lipid/polymeric NP (TPGS-PLGA)	EPR (injured tissue targeting), TTP (mito-targeting)	Tanshinone	Pleiotropic [48]
Lipid/polymeric NPs (MCTD)	EPR effect (injured tissue targeting), IMTP (cell targeting), SS31 (mito-targeting)	Resveratrol	Pleiotropic [49]
Liposome (mito-porter)	R8 (cell targeting), fusogenic lipid (mito-targeting)	Multiple mitoprotective agents	Pleiotropic [50,51,52,53]

Abbreviations: CD44, extracellular matrix protein receptor; CsA, cyclosporine A; EPR, enhanced permeability and retention; HA, hyaluronic acid; IMPT, ischemic myocardium-targeting peptide MCTD, multistage continuous targeted drug delivery; MPTPO, mitochondrial permeability transition pore opening; NP, nanoparticle; PEG, polyethylene glycol; PLGA, poly(lactic-co-glycolic); PTM, post-translational protein modification; R8, octa-arginine peptide; RGD, arginyl-glycyl-aspartic acid; ROS, reactive oxygen species; SS31, Szeto–Schiller-tetra-peptide 31; TPGS, d-α-tocopheryl-PEG-succinate; TTP, triphenylphosphonium.

**Table 2:** Mitochondrial delivery of cardioprotective NO and H_2_S donors and putative best therapeutic indications to be explored in humans.

Carrier	Function	Cardioprotective Mechanism	Therapeutic Indications
TTP-conjugation MitoSNO	NO donor	Reversible mitochondria protein nitrosilation	Cardiac IR Post ischemic HF
TTP-conjugation AP39	H_2_S donor	MTPT opening inhibition	Cardioplegic solution Cardiac transplantation HF
4CPI	H_2_S donor	MitoK-ATP activation	Cardioplegic solution Cardiac transplantation HF
3PI	H_2_S donor	MitoK-ATP activation	Cardioplegic solution Cardiac transplantation HF

Abbreviations: AP39, [(10-oxo-10-(4-(3-thioxo-3H-1,2-dithiol-5yl)phenoxy)decyl) triphenylphosphonium bromide]; 4CPI, 4-carboxy-phenyl-isothiocyanate; HF, heart failure; MitoK-ATP, ATP sensitive mitochondrial potassium channel; 3PI, 3-pyridyl-isothiocyanate; TTP, triphenylphosphonium.
